# HtrA1 expression associated with the occurrence and development of esophageal cancer

**DOI:** 10.1186/1477-7819-10-179

**Published:** 2012-08-30

**Authors:** Youtao Yu, Wenlong Shao, Yi Hu, Jingyan Zhang, Hao Song, Zhi-hua Zhu

**Affiliations:** 1 Department of Interventional Radiology, The Third Affiliated Hospital of Harbin Medical University, No. 150, Haping Rd, Harbin, 150040, China; 2 Department of Cardiothoracic Surgery, The First Affiliated Hospital of Guangzhou Medical College, Guangzhou, China; 3 Guangzhou Institute of Respiratory Disease & China State Key Laboratory of Respiratory Disease, Guangzhou, China; 4 State Key Laboratory of Oncology in South China, Guangzhou, China; 5 Department of Thoracic Surgery, Cancer Center, Sun Yat-sen University, Guangzhou, China

**Keywords:** Esophageal cancer, HtrA1 expression, Semi-quantitative RT-PCR, SiRNA

## Abstract

**Background:**

The purposes of this study were to measure both the mRNA and protein expression levels of high-temperature requirement serine peptidase 1 (HtrA1) in human esophageal cancer tissues and their adjacent, comparatively normal esophageal tissues.

**Methods:**

The expression levels of HtrA1 mRNA and protein in both tissue types were measured by semi-quantitative RT-PCR (reverse transcription-polymerase chain reaction) and Western blotting. The clinical and pathological correlation between HtrA1 expression levels and the occurrence and development of esophageal cancer was analyzed.

**Results:**

The expression levels of HtrA1 mRNA and protein in esophageal carcinoma were significantly lower than the levels expressed in their adjacent normal esophageal tissue (*p* < 0.05). The more highly undifferentiated esophageal tumor cells expressed lower HtrA1 mRNA and protein expression levels (*p* < 0.05). Patients with tumors in early pathological stages (I-II) had significantly higher HtrA1 mRNA and protein expression levels than did patients with tumors in mid-to-late pathological stages (III-IV) (*p* < 0.05). Patients with positive lymph node metastasis had significantly lower HtrA1 mRNA and protein expression levels than did patients with lymph node-negative disease (*p* < 0.05).

**Conclusions:**

HtrA1 expression is associated with the occurrence and development of esophageal cancer.

## Background

Multimodality therapy for esophageal cancer has improved patient outcomes and is favored by the current standard of practice for surgical patients [1-3]. Neoadjuvant chemoradiotherapy followed by surgery appears to increase resectability, produce tumor downstaging and improve local control, disease-free survival and overall survival compared to surgery alone [4-7]. However, esophageal cancers have a poor prognosis and a 5-year survival rate of less than 10% [8,9]. This poor outcome is mainly due to frequent and extensive invasion outside of the esophagus or regional lymph node metastasis, which can occur during the early stages of esophageal cancer [10,11]. The occurrence and development of esophageal cancers is the result of the cooperative action of multiple genes. Therefore, searching for genes associated with the occurrence and development of esophageal cancer and its metastasis has become a heavily investigated topic in current studies. Examining the genes associated with the occurrence, development and metastasis of esophageal cancer may provide a theoretical foundation and potential therapeutic targets for the treatment of metastatic esophageal cancer.

The mammalian HtrA serine protease family is composed of homologous serine proteases with different domains [12]. HtrA, also known as DegP, is a membrane serine protease with properties similar to those of heat shock proteins. HtrA is widely expressed in various microorganisms, plants and animals [13,14]. HtrA acts as a molecular chaperone at low temperatures, whereas at high temperatures, HtrA functions as a serine protease involved in cellular defense against various stress conditions, such as heat shock, oxidative stress, inflammation, ischemia/reperfusion and cancer, and HtrA degrades misfolded proteins within the cytoplasm [15-17]. Currently, four members have been reported as belonging to the human HtrA family: HtrA1, HtrA2, HtrA3 and HtrA4 [18]. HtrA1 was the first member identified in the human HtrA serine protease protein family. HtrA1 is a secreted protein that is involved in the degradation of the extracellular matrix. Initially detected in fibroblasts infected with simian virus 40 (SV40), HtrA1 was also later found in cases of cartilage arthritis in which it played a positive role in regulating osteoarthritis [19,20].

HtrA1 is considered to be a tumor suppressor gene that reduces the transforming ability of fibroblasts and suppresses the growth of highly invasive tumors, such as ovarian cancer and invasive melanoma [15,20,21]. In addition, previous research has reported that HtrA1 protein expression is associated with tumor migration and metastasis [22,23]. The expression of HtrA1 in human esophageal cancer tissues, as well as its relevance in the occurrence and development of esophageal cancers, has not yet been reported. Also, there have been no reports correlating HtrA1 expression with esophageal cancer cell metastasis. This study utilized semi-quantitative RT-PCR (reverse transcription-polymerase chain reaction) and Western blotting to measure HtrA1 mRNA and protein expression in human esophageal cancer tissues and their adjacent normal esophageal tissues. We also used RNA interference or transfected an HtrA1 recombinant plasmid to downregulate or overexpress the HtrA1 protein in the Eca-109 human esophageal cancer cell line. Subsequent changes in the invasiveness of Eca-109 cells were observed, and the relationship between HtrA1 protein expression and the occurrence, development and metastasis of human esophageal cancer was explored.

## Methods

### Specimens

Fresh specimens from 63 cases of esophageal cancers treated in the Third Affiliated Hospital of Harbin Medical University from June 2004 to June 2010 (50 males, 13 females) were surgically removed and immediately stored in liquid nitrogen for future use. No patients received preoperative chemotherapy or radiotherapy, nor did any patients have histories of other treatments. Also, the tumors were not associated with other inflammatory diseases. The pathology department in our hospital confirmed all 63 cases of surgically resected esophageal cancers and their adjacent normal esophageal tissues (more than 5 cm away from the cancerous tissue). The ages of the patients ranged from 45–79 years with a mean age of 73.42 years. There were 19 cases younger than 60 years and 44 cases equal to or older than 60 years. The primary tumor was smaller than 3 cm in 36 cases and greater than or equal to 3 cm in 27 cases. In terms of differentiation, 34 cases displayed high or intermediate differentiation (17 cases high differentiation of squamous cell carcinoma and 11 cases intermediate differentiation of squamous cell carcinoma), whereas 29 cases displayed poor differentiation (26 cases squamous cell carcinoma). There were 33 cases of pathological stage I-II disease and 30 cases of stage III-IV disease. Thirty-seven cases had lymph node metastasis, whereas 26 cases had no lymph node involvement. Distant metastasis was identified in 32 cases and was absent in 31 cases.

### Reagents

To detect HtrA1 mRNA expression, the upstream primer P1 for HtrA1 (GenBank no.: NM_002775.4) was 5'-TGG ACG GTG AAG TGA TTG G-3', and the downstream primer P2 was 5'-AGC TCA TGC CTC TGC CTA T-3'. The size of expected amplification product was 455 base pairs (bp). According to the sequence of the human HtrA1 mRNA in GenBank and an analysis of its restriction digestion sites, we used the primer premier 5.0 software to design a pair of primers to amplify the HtrA1 open reading frame (ORF). The upstream primer used for HtrA1 amplification was 5'-CG GGATCC ATG CAG ATC CCG CGC GCC GC-3', which contains a BamHI restriction site. The downstream primer used for HtrA1 amplification was 5'-CC CTCGAG TGG GTC AAT TTC TTC GGG AA-3', which contains an XhoI restriction site. The upstream primer for the internal reference, GAPDH (GenBank no.: NM_002046), was 5'-CCA CAG TCC ATG CCA TCA CT-3', and the downstream primer for GAPDH was 5'-TCC ACC ACC CTG TTG CTG TAG-3'. The expected amplification product was 451 bp. All of the above primers were synthesized by the Shanghai Invitrogen Biotechnology Company.

The primary antibody against HtrA1was a rabbit anti-human polyclonal antibody, and the primary antibody against β-actin was a mouse anti-human monoclonal antibody. Both primary antibodies were purchased from Abcam, UK. The secondary antibodies were IRDye 800 conjugated, affinity-purified, goat anti-mouse IgG and IRDye 800 conjugated, affinity-purified, goat anti-rabbit IgG, both of which were purchased from the Odyssey Corporation.

The vectors (pcDNA3.1 and pGEM-T) and the Trizol total RNA extraction kit were purchased from Invitrogen, USA. The restriction enzymes BamHI and XhoI and the DNA size marker were purchased from TaKaRa, Japan. The reverse transcription kit was purchased from Qiagen, Germany. The T4 DNA ligation kit was purchased from Promega, USA. Taq DNA polymerase and pre-stained protein molecular weight standards were purchased from Fermentas, USA. The HtrA1 siRNA (5'-Fluo-CGGCCGAAGUUGCCUCUUUTT-3') and the negative control siRNA (5'-Fluo-UCCUGCUGGAGCCUCAUGUTT-3') were purchased from Sigma Aldrich, USA. The Eca-109 human esophageal cancer cell line was purchased from the Shanghai Institute of Cell Biology, Chinese Academy of Sciences. RPMI 1640, trypsin, fetal bovine serum and the Lipofectamine 2000 transfection reagent were all purchased from Invitrogen, USA. Tissue culture plates and the Transwell invasion chamber were purchased from the Corning Corporation.

### RT-PCR

One hundred milligrams of esophageal cancer tissue or its adjacent normal esophageal tissue (more than 5 cm from the cancer) were collected separately and homogenized in1 ml of Trizol reagent. Total RNA was extracted in accordance with the procedures outlined in the Trizol product manual. The reverse transcription kit was used to perform the reverse transcription reaction. The first-strand cDNA was synthesized according to the product manual and stored at −20°C for future use.

The HtrA1 and GAPDH primers were synthesized by the Shanghai Invitrogen Biotechnology Company, dissolved in ddH_2_O and stored at −20°C for future use. One microliter of cDNA was added to a 25-μl PCR reaction. The amplification conditions consisted of an initial denaturation for 5 min at 94°C followed by 30 cycles of amplification with denaturation for 30 s at 94°C, annealing for 30 s at 56°C, extension for 30 s at 72°C and a final extension for 10 min at 72°C. PCR products were verified by electrophoresis on a 1.5% agarose gel. The relative HtrA1 mRNA content was determined by the ratio of HtrA1 to GAPDH intensity, which was calculated using the QuantityOne software (Bio-Rad, USA).

### Western blotting

Fifty milligrams of tissue sample that had been frozen in liquid nitrogen was ground and homogenized in 1 ml of RIPA lysis buffer [150 mM NaCl, 1% NP40, 0.5% sodium deoxycholate, 0.1% SDS, 50 mM Tris (pH 7.9), 10 mM NaF, PMSF and 1 × protease inhibitors (Complete cocktail tablets, Roche)]. The homogenate was transferred to a 1.5-ml centrifuge tube and centrifuged at 16,000 × g for 30 min. The concentration of the total protein in the supernatant was measured using the BCA Protein Assay Kit (Shanghai Biocolor BioScience & Technology Co.). Ten micrograms of total protein from each esophageal cancer case was mixed together and used as a single combined esophageal cancer tissue protein sample. The same was done to create the adjacent normal esophageal protein sample.

A polyacrylamide gel consisting of a 5% stacking gel and a 12% separating gel was cast. A total of 50 μg of protein was loaded per lane, separated by electrophoresis and transferred to a PVDF membrane via the wet transfer method (Bio-Rad, USA). After blocking in a solution of 5% non-fat milk in TBST [10 mM Tris–HCl (pH7.5), 150 mM NaCl and 0.1% Tween-20] at room temperature for 1 h, a rabbit polyclonal anti-human HtrA1 antibody (1:500 dilution) or a mouse anti-human β-actin monoclonal antibody (1:1,000 dilution) was applied to the blot and incubated at 4°C overnight. The appropriate IRDye 800 labeled secondary antibody (1:2000 dilution in PBS) was added and incubated at 4°C overnight. After washing with TBST, the membrane was scanned with the Odyssey Infrared Imaging System (Rockland Co.). The relative HtrA1 protein content was determined using the ratio of HtrA1 intensity to β-actin intensity, which was analyzed using the QuantityOne software (Bio-Rad, USA).

### Immunohistochemistry

The 4% paraformaldehyde-fixed tumor tissue was paraffin embedded, cut into 5-μm serial sections and blocked with normal goat serum at room temperature for 20 min. The tissue section was probed with the HtrA1 antibody (at a working dilution of 1:200) at 4°C overnight and then washed three times with PBS for 2 min each. The section was subsequently probed with the biotinylated goat anti-rabbit IgG secondary antibody (at a working dilution of 1:150) at 4°C overnight and washed three times with PBS for 2 min. The section was stained by SAB at 37°C for 20 min, was washed with PBS four times for 5 min and then was incubated in DAB at room temperature for 10 min. The section was stained by hematoxylin and observed using a Leica microscope. Yellow and brown staining in the immunohistochemical results was considered positive.

### Transfection of pcDNA3.1-HtrA1 and HtrA1 siRNA into Eca-109 cells and measurement of cell invasiveness and metastasis

PCR amplification was performed using the first strand cDNA derived from the adjacent normal esophageal tissue as the template. The HtrA1 upstream and downstream primers were used for the PCR amplification. The PCR product was ligated into the pGEM-T vector. After confirming proper ligation by restriction enzyme digestion, the properly constructed recombinant plasmid was sent to Shanghai Invitrogen Biotechnology Co., Ltd., for DNA sequencing. After sequence verification, both the recombinant plasmid pGEM-HtrA1 and the pcDNA3.1 vector were subjected to a BamHI and XhoI double digest. The digested products were purified, and the HtrA1 gene was ligated into the pcDNA3.1 vector using T4 DNA ligase. The recombinant plasmid, pcDNA3.1-HtrA1, was transformed to DH5α competent cells to amplify and isolate the construct.

Eca-109 cells were seeded into six-well tissue culture plates at a concentration of 1 × 10^6^ cells per well. After an overnight incubation, the recombinant pcDNA3.1-HtrA1 plasmid and the sense or antisense HtrA1 siRNAs were transfected into Eca-109 cells using Lipofectamine 2000. A Western blot was used to detect the changes in the HtrA1 protein expression levels in each group of cells to verify the effect of RNA interference or the overexpression of HtrA1. A Transwell chamber invasion assay was used to measure changes in the invasiveness of the Eca-109 cells between the untransfected control group, the empty vector-transfected control group, the HtrA1 siRNA-transfected group and the recombinant plasmid pcDNA3.1-HtrA1-transfected group. The number of cells crossing the Transwell polycarbonate membrane was counted using a Leica microscope. Cells that crossed the polycarbonate membrane were considered to be invasive. A total of eight fields were randomly observed.

### Statistical analyses

The Stata 7.0 statistical software was used for statistical analyses of the experimental results. The statistical methods used were the chi-squared (χ^2^) test and Student’s *t* test. A *p*-value of less than 0.05 was considered to be statistically significant.

## Results

### RT-PCR detection of HtrA1 mRNA expression in esophageal carcinoma tissue

**Figure 1:**
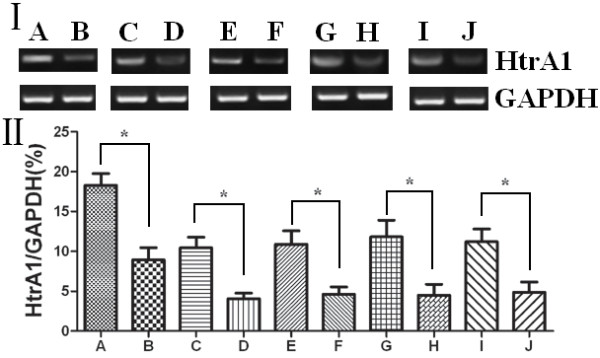
**I: HtrA1 mRNA expression in esophageal cancers and their adjacent normal esophageal tissues and its relation to clinicopathological factors.** II: The relative changes of HtrA1 mRNA expression in esophageal carcinomas and their adjacent normal esophageal tissues. (**A**) Adjacent normal esophageal tissue; (**B**) esophageal cancer tissue; (**C**) highly and intermediately differentiated; (**D**) poorly differentiated; (**E**) early pathological stage (I-II) tumors; (**F**) mid-to-late pathological stage (III-IV) tumors; (**G**) lymph node metastasis negative; (**H**) lymph node metastasis positive; (**I**) distant metastasis negative; (**J**) distant metastasis positive. *p < 0.05.

The RT-PCR produced amplified products of the expected sizes (i.e., 455 bp and 451 bp for HtrA1 and the internal reference GAPDH, respectively). The PCR was followed by DNA isolation, cloning and sequencing. The percentage of positive HtrA1 expression in human esophageal cancer tissues and their adjacent normal esophageal tissues was 42.86% and 68.25%, respectively. HtrA1 mRNA expression in the esophageal cancer tissues was significantly lower than in their adjacent normal esophageal tissue (*p* < 0.05). More highly undifferentiated esophageal cells displayed lower HtrA1 mRNA expression levels (*p* < 0.05). The HtrA1 mRNA expression in tumors of early pathological stages (I-II) was significantly higher than in tumors of mid-to-late pathological stages (III-IV) (*p* < 0.05). Patients with positive lymph node metastasis had significantly lower HtrA1 mRNA expression levels than did patients with negative lymph node metastasis (*p* < 0.05). Patients with positive distant metastasis had significantly lower HtrA1 mRNA expression than did patients without distant metastasis (*p* < 0.05) (Figure 1). HtrA1 mRNA expression was not associated with a patient’s gender, age or tumor size (*p* > 0.05), as displayed in Table 1.

**Table 1:** HtrA1 mRNA expression in esophageal cancer tissue and its relationship with clinicopathological factors

**Pathologic parameter**	**Cases**	**HtrA1-positive rate (%)**	***χ***^**2**^	***P***
Normal tissue	63	43 (68.25)	8.2286	0.004*
Carcinoma tissue	63	27 (42.86)		
Sex				
Male	50	23 (46.00)	0.9773	0.323
Female	13	4 (30.77)		
Age				
< 60	19	6 (31.58)	1.4130	0.235
≥ 60	44	21 (47.73)		
Size of primary carcinoma (cm)				
< 3	36	15 (41.67)	0.0486	0.825
≥ 3	27	12 (44.44)		
Degree of differentiation				
Well and moderately differentiated	34	19 (55.88)	5.1169	0.024*
Poorly differentiated	29	8 (27.59)		
Pathological stage				
I − II	33	19 (57.58)	6.1303	0.013*
III − IV	30	8 (26.67)		
Lymph nodes metastasis				
Negative	26	17 (65.38)	9.1739	0.002*
Positive	37	10 (27.03)		
Distant metastasis				
Negative	31	21 (67.74)	15.4325	0.000*
Positive	32	6 (18.75)		

### Western blot detection of HtrA1 protein expression in esophageal cancer

**Figure 2:**
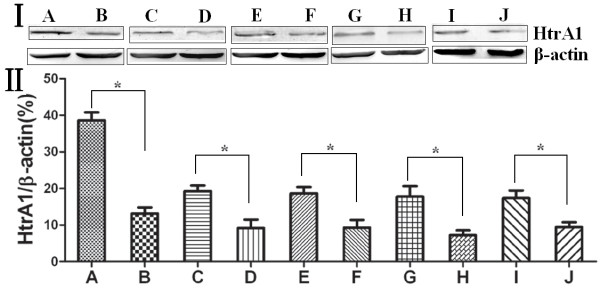
**I: HtrA1 protein expression in esophageal cancer and the adjacent normal esophageal tissues and its relationship with clinicopathological factors.** II: The relative changes of HtrA1 protein expression in esophageal carcinoma and its adjacent normal esophageal tissues. (**A**) Adjacent normal esophageal tissue; (**B**) esophageal cancer tissue; (**C**) highly and intermediately differentiated; (**D**) poorly differentiated; (**E**) early pathological stage (I, II) patients; (**F**) mid-to-late pathological stage (III, IV) patients; (**G**) lymph node metastasis negative; (**H**) lymph node metastasis positive; (**I**) distant metastasis negative; (**J**) distant metastasis positive. *p < 0.05.

Western blot analysis revealed that HtrA1 protein expression in esophageal cancer tissue was significantly lower than in the adjacent normal esophageal tissue (*p* < 0.05). Indeed, the more undifferentiated esophageal cells displayed lower HtrA1 protein expression levels (*p* < 0.05). Early pathological stage (I-II) tumors had significantly higher HtrA1 protein expression than did the mid-to-late pathological stage (III-IV) tumors (*p* < 0.05). Patients with positive lymph node metastasis had significantly lower HtrA1 protein expression levels than did patients with lymph node-negative disease (*p* < 0.05). Patients with positive distant metastasis had significantly lower HtrA1 protein expression levels compared to patients with distant metastasis negative disease (*p* < 0.05). The results are shown in Table 2 and Figure 2.

**Table 2:** HtrA1 protein expression in esophageal cancer tissue

**Pathologic parameter**	**Cases**	**Gray-scale ratio of HtrA1/β-actin**	***P***
Normal tissue	63	38.60 ± 3.81	<0.05
Carcinoma tissue	63	13.13 ± 2.90	
Degree of differentiation			
Well and moderately differentiated	34	19.2747 ± 2.65	<0.05
Poorly differentiated	29	9.17 ± 3.97	
Pathological stage			
I − II	33	18.63 ± 3.05	<0.05
III − IV	30	9.30 ± 3.66	
Lymph nodes metastasis			
Negative	26	17.79 ± 4.96	<0.05
Positive	37	7.24 ± 2.31	
Distant metastasis			
Negative	31	17.37 ± 3.60	<0.05
Positive	32	9.43 ± 2.30	

### Immunohistochemical detection of HtrA1 protein in esophageal cancer and the adjacent normal esophageal tissue

**Figure 3:**
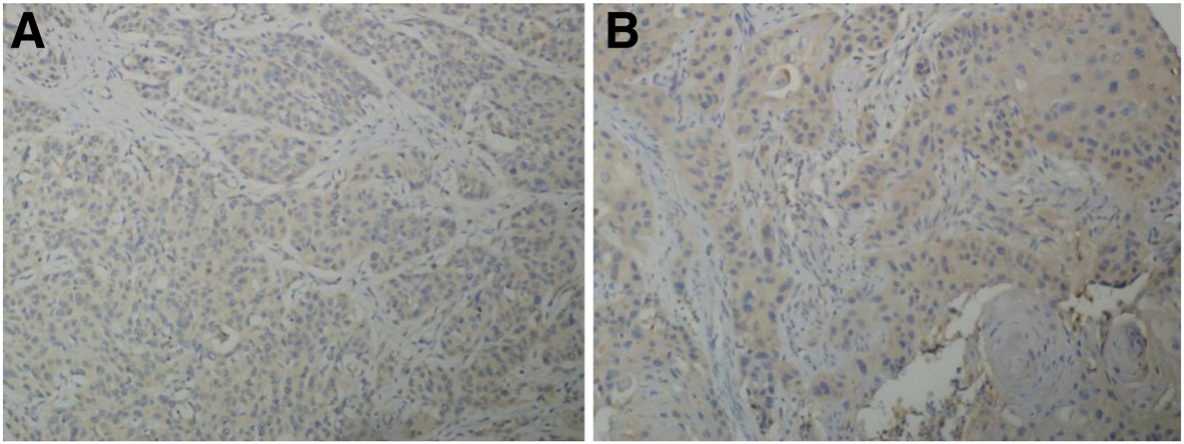
**HtrA1 expression in esophageal cancer and the adjacent normal esophageal tissue.** (**A**) Adjacent normal esophageal tissue; (**B**) esophageal cancer.

HtrA1 staining was mainly localized in the cytoplasm, and no positive staining was detected in the nucleus. HtrA1 protein expression in esophageal cancer was significantly lower than that in adjacent normal esophageal tissues (Figure 3).

### The effect of HtrA1 expression levels on the *in vitro* invasiveness of the Eca-109 human esophageal cancer cell line

**Figure 4:**
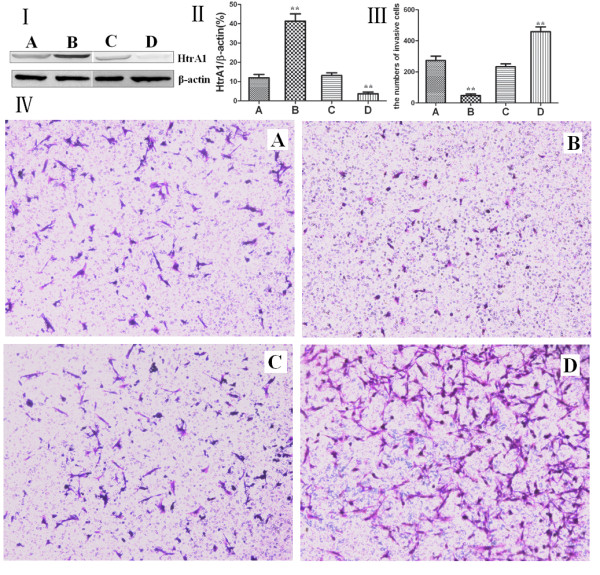
**I: Western blot analysis of HtrA1 protein expression for each group of Eca-109 cells.** II: The relative changes of the HtrA1 protein expression levels in each group. III: Measurement of the number of cells crossing the Transwell invasion chamber. IV: Bright field microscopy of each group of Eca-109 cells stained with crystal violet. (**A**) The untransfected control group; (**B**) the pcDNA3.1-HtrA1-transfected group; (**C**) the control siRNA-transfected group; (**D**) the HtrA1 siRNA-transfected group. **p < 0.01.

We successfully constructed the recombinant pcDNA3.1-HtrA1plasmid and used Lipofectamine 2000 to transfect the plasmid into Eca-109 cells to overexpress the HtrA1 protein. Additionally, HtrA1 protein levels were reduced by RNA interference. The changes in the invasiveness of the Eca-109 human esophageal cancer cells were tested using a Transwell invasion chamber. Eca-109 cells were divided into four groups: the untransfected control group, the recombinant plasmid pcDNA3.1-HtrA1-transfected group, the siRNA control-transfected group and the HtrA1 siRNA-transfected group. Western blot analysis revealed that HtrA1 protein expression levels were significantly increased in the Eca-109 cells transfected with pcDNA3.1-HtrA1 (*p* < 0.01) (Table 3). The HtrA1-overexpressing cells also demonstrated a decrease in the number of cells (47 cells/field) crossing the polycarbonate membrane of the Transwell invasion chamber (*p* < 0.01). HtrA1 protein expression levels were significantly decreased in the group of Eca-109 cells transfected with the HtrA1 siRNA (*p* < 0.01). The knockdown cells displayed a significantly increased number of cells (459 cells/field) crossing the polycarbonate membrane of the Transwell invasion chamber relative to the control (*p* < 0.01). The untransfected group and the transfected control groups of the Eca-109 cells displayed no significant difference in HtrA1 protein expression or cell invasiveness (Figure 4). These data suggest that an increased level of HtrA1 protein can reduce the invasiveness of t Eca-109 cells, whereas a reduced level of HtrA1 protein promotes Eca-109 cell invasion. This finding also indicates that HtrA1 protein expression levels are negatively correlated with Eca-109 cell invasiveness.

**Table 3:** The comparison of invasiveness of Eca-109 cells in relation to expression of HtrA1

**Groups**	**Gray-scale ratio of HtrA1/β-actin**	**Number of invasive cells**
Untransfected control group	11.99 ± 3.05	273.33 ± 48.44
pcDNA3.1-HtrA1-transfected group	41.34 ± 6.52**	47.67 ± 17.24**
Control siRNA-transfected group	13.10 ± 2.48	233.00 ± 33.18
HtrA1 siRNA-transfected group	3.65 ± 1.57**	459.33 ± 51.73**

**p < 0.01 versus the untransfected control group.

## Discussion

HtrA1 was the first identified member of the HtrA serine protease family. Structurally, HtrA1 has two isolated homology regions. The C-terminal region contains the highly conserved insulin serine protease (SP) region and the PDZ region, which are regions specific to the HtrA family. The N-terminal region contains domains homologous to the insulin-like growth factor binding protein (IGFBP) and follistatin (FS). Between the IGFBP region and the SP region, there is a region similar to the protease inhibitor Kazal [18,24-26].

Many studies have demonstrated that the HtrA1 serine protease is involved in a variety of diseases [27-29]. A single nucleotide polymorphism (SNP), rsll200638, was identified in the HtrA1 gene promoter and found to be significantly correlated with age-related macular degeneration (AMD) with a population-attributable risk of 49.3%. Individuals with the HtrA1 polymorphism have a ten-fold greater risk of developing AMD [28]. HtrA1 can bind to and inhibit transforming growth factor β (TGF-β), which is an important regulator of the extracellular matrix deposition and angiogenesis [30]. De Luca et al. have confirmed that the HtrA1 serine protease is associated with gestational hypertension and that HtrA1 expression is significantly increased in late pregnancy, with higher expression levels being observed in the syncytiotrophoblast versus the cytotrophoblast [31].

HtrA1 is considered to be a tumor suppressor gene [15,20,21]. In 2006, Bowden et al. used RT-PCR, Western blotting and immunohistochemistry to detect the gene and protein expression levels of HtrA1 and HtrA3 in normal human endometrium and endometrial carcinoma. Their results illustrated that HtrA1 mRNA levels in endometrial carcinoma were significantly lower than in the normal endometrium. Additionally, as the endometrial cancer histological grade (G1, G2, G3) increased, there was clearly a greater decrease in HtrA1 mRNA levels. Immunohistochemistry revealed that HtrA1 expression in histological grade G3 tumors was significantly lower than in the G1 grade [32].

In 2008, Joanna Narkiewicz et al. used semi-quantitative RT-PCR and Western blotting to measure HtrA1, HtrA2 and HtrA3 mRNA and protein expression levels in ovarian cancers. The results revealed that HtrA1 mRNA expression in ovarian cancer was significantly decreased compared with normal ovarian tissue [33]. Zhu et al. confirmed that HtrA1 expression in liver cancer tissues was significantly lower than in their adjacent liver tissue and that HtrA1 was associated with the occurrence and development of liver cancer [23]. De Luca et al. confirmed the expression of HtrA1 in normal esophageal tissues [34]. In the present study, we showed that the percentages of positive HtrA1 expression in human esophageal cancer tissues and their adjacent normal tissues were 42.86% and 68.25%, respectively. Also, HtrA1 mRNA and protein expression levels in esophageal carcinoma were significantly lower than in the adjacent normal esophageal tissue (*p* <0.05). The more highly undifferentiated esophageal cells displayed lower HtrA1 mRNA and protein expression levels (*p* < 0.05). Patients with early pathological stage tumors (I-II) had significantly higher HtrA1 mRNA and protein expression levels than in patients with mid-to-late pathological stage tumors (III-IV) (*p* < 0.05). Patients with positive lymph node metastasis had significantly lower HtrA1 mRNA and protein expression levels versus patients with lymph node-negative disease (*p* < 0.05). Patients with positive distant metastasis had significantly lower HtrA1 mRNA and protein expression levels than patients with no distant metastasis (*p* < 0.05). Finally, HtrA1 mRNA and protein expression levels were not associated with a patient’s gender, age or tumor size (*p* > 0.05). Our results are consistent with previous studies.

Mullany et al. have reported that downregulating HtrA1 expression in Hec1A and Hec1B cells (both of which are endometrial carcinoma cell lines) via RNA interference leads to a three- to four-fold increase in the invasiveness of these cells, whereas overexpressing HtrA1in Ark1 and Ark2 cells leads to a three- to four-fold decrease in their invasiveness [35]. Chien et al. also confirmed that downregulating HtrA1 can promote cell invasion, that stimulating HtrA1 can reduce cell invasiveness and that HtrA1 is a microtubule-associated protein that regulates cell motility by regulating the stability of microtubules [36].

Many reports indicate that during the early stages of tumorigenesis (when the tumor is still benign), TGF-β1 acts a tumor suppressor gene; however, in the later stages of tumorigenesis, TGF-β1 becomes a promoter for tumor progression, invasion and metastasis [37]. HtrA1 can bind to and transform TGF-β family members, leading to the inhibition of TGF-β signaling. The proteolytic function of HtrA1 is essential for this inhibitory effect [38]. In this study, we successfully transfected Eca-109 cells with the pcDNA3.1-HtrA1 recombinant expression plasmid or an HtrA1 siRNA. We observed changes in cell invasiveness in these lines using a Transwell assay. Eca-109 cells transfected with the pcDNA3.1-HtrA1 recombinant plasmid displayed a significant increase in HtrA1 protein expression levels (*p* < 0.01) and a significantly decreased number of cells crossing the Transwell chamber relative to the untransfected control group and the empty vector-transfected control group (*p* < 0.01). The Eca-109 cells transfected with the HtrA1 siRNA displayed significantly lower HtrA1 protein expression levels (*p* < 0.01) and significantly higher numbers of cells crossing the Transwell chamber relative to the untransfected control group and the non-targeting siRNA transfected control group (*p* < 0.01). These results are consistent with those of previous studies.

## Conclusion

HtrA1 protein expression is associated with the occurrence and development of esophageal cancer. HtrA1 participates in the invasion and metastasis of esophageal cancer cells. The underlying mechanism of this process may be related to the TGF-β cell-signaling pathway, but the exact mechanism requires further elucidation. In the future, HtrA1 may be a potential target for the treatment of esophageal cancer.

## Competing interests

The authors declare no competing interest.

## Authors’ contributions

YY: Design, acquisition of data, analysis and interpretation of data, drafting of manuscript, critical revision, final approval. WS: Design, drafting of manuscript, critical revision, final approval. YH: Design, acquisition of data, critical revision, final approval. JZ: Design, acquisition of data, critical revision, final approval. HS: Design, acquisition of data, analysis and interpretation of data, drafting of manuscript, critical revision, final approval. ZZ: Design, acquisition of data, analysis and interpretation of data, drafting of manuscript, critical revision, final approval. All authors read and approved the final manuscript.

## References

[B1] Feng XX Duan PF Wang LB Lu ZX Polymorphisms of XPC gene and susceptibility of esophageal cancer Chin J Cancer Res 2010 22 49 54 10.1007/s11670-010-0049-0

[B2] Nasr JY Schoen RE Prevalence of adenocarcinoma at esophagectomy for Barrett’s esophagus with high grade dysplasia J Gastrointest Oncol 2011 2 34 38 22811825 10.3978/j.issn.2078-6891.2010.027 PMC3397590

[B3] Wagner TD Khushalani N Yang GY Clinical T2N0M0 carcinoma of thoracic esophagus J Thorac Dis 2010 2 36 42 22263015 PMC3256438

[B4] Jabbour SK Thomas CR Radiation therapy in the postoperative management of esophageal cancer J Gastrointest Oncol 2010 1 102 111 22811814 10.3978/j.issn.2078-6891.2010.013 PMC3397583

[B5] Das P Esophageal cancer: is preoperative chemoradiation the new standard? J Gastrointest Oncol 2010 1 68 69 22811807 10.3978/j.issn.2078-6891.2010.014 PMC3397580

[B6] Kleinberg L Does postoperative radiation therapy benefit patients with esophageal cancer? J Gastrointest Oncol 2010 1 70 71 22811808 10.3978/j.issn.2078-6891.2010.015 PMC3397577

[B7] Prasanna PGS Stone HB Wong RS Capala J Bernhard EJ Vikram B Coleman CN Normal tissue protection for improving radiotherapy: where are the gaps? Transl Cancer Res 2012 1 35 48 22866245 PMC3411185

[B8] Hopkins S Yang GY Positron emission tomography's utility in esophageal cancer management J Thorac Dis 2009 1 29 33 22262999 PMC3256486

[B9] Chen YJ Kernstine KH Shibata S Lim D Smith DD Tang M Liu A Pezner RD Wong JY Image-guided radiotherapy of esophageal cancer by helical tomotherapy: acute toxicity and preliminary clinical outcome J Thorac Dis 2009 1 11 16 22262996 PMC3256488

[B10] Patnaik SK Mallick R Yendamuri S MicroRNA’s and esophageal cancer J Gastrointest Oncol 2010 1 55 63 22811805 10.3978/j.issn.2078-6891.2010.011 PMC3397565

[B11] Yu C Fan S Sun Y Pickwell-Macpherson E The potential of terahertz imaging for cancer diagnosis: a review of investigations to date Quant Imaging Med Surg 2012 2 33 45 10.3978/j.issn.2223-4292.2012.01.04 PMC3496499 23256057

[B12] Clausen T Kaiser M Huber R Ehrmann M HTRA proteases: regulated proteolysis in protein quality control Nat Rev Mol Cell Biol 2011 12 152 162 21326199 10.1038/nrm3065

[B13] Schuhmann H Adamska I Deg proteases and their role in protein quality control and processing in different subcellular compartments of the plant cell Physiol Plant 2012 145 224 234 10.1111/j.1399-3054.2011.01533.x 22008015

[B14] Backert S Clyne M Pathogenesis of Helicobacter pylori infection Helicobacter 2011 16 Suppl 1 19 25 21896081 10.1111/j.1523-5378.2011.00876.x

[B15] Zurawa-Janicka D Skorko-Glonek J Lipinska B HtrA proteins as targets in therapy of cancer and other diseases Expert Opin Ther Targets 2010 14 665 679 10.1517/14728222.2010.487867 20469960

[B16] Huesgen PF Schuhmann H Adamska I Deg/HtrA proteases as components of a network for photosystem II quality control in chloroplasts and cyanobacteria Res Microbiol 2009 160 726 732 10.1016/j.resmic.2009.08.005 19732828

[B17] Meltzer M Hasenbein S Mamant N Structure, function and regulation of the conserved serine proteases DegP and DegS of Escherichia coli Res Microbiol 2009 160 660 666 10.1016/j.resmic.2009.07.012 19695325

[B18] Zurawa-Janicka D Narkiewicz J Lipinska B Characterization of the HtrA family of proteins Postepy Biochem 2007 53 27 36 17718385

[B19] Chen W Xu W Tao Q Meta-analysis of the association of the HTRA1 polymorphisms with the risk of age-related macular degeneration Exp Eye Res 2009 89 292 300 10.1016/j.exer.2008.10.017 19026638

[B20] Canfield AE Hadfield KD Rock CF HtrA1: a novel regulator of physiological and pathological matrix mineralization? Biochem Soc Trans 2007 35 669 671 10.1042/BST0350669 17635117

[B21] Chien J Campioni M Shridhar V HtrA serine proteases as potential therapeutic targets in cancer Curr Cancer Drug Targets 2009 9 451 468 10.2174/156800909788486704 19519315 PMC3905973

[B22] Mullany SA Moslemi-Kebria M Rattan R Expression and functional significance of HtrA1 loss in endometrial cancer Clin Cancer Res 2011 17 427 436 10.1158/1078-0432.CCR-09-3069 21098697 PMC3057564

[B23] Zhu F Jin L Luo TP Serine protease HtrA1 expression in human hepatocellular carcinoma Hepatobiliary Pancreat Dis Int 2010 9 508 512 20943460

[B24] Chien J He X Shridhar V Identification of tubulins as substrates of serine protease HtrA1 by mixture-based oriented peptide library screening J Cell Biochem 2009 107 253 263 10.1002/jcb.22121 19301262 PMC2983094

[B25] Zhu YJ Palliative radiotherapy for painful bone metastases: short-course or long-course? Ann Palliat Med 2011 1 78 80 10.3978/j.issn.2224-5820.2011.10.03 25841432

[B26] Gray CW Ward RV Karran E Characterization of human HtrA2, a novel serine protease involved in the mammalian cellular stress response Eur J Biochem 2000 267 5699 5710 10.1046/j.1432-1327.2000.01589.x 10971580

[B27] Zhang L Lim SL Du H HTRA1 regulates angiogenesis through TGF-beta family member GDF6 J Biol Chem 2011 10.1074/jbc.M111.275990 PMC3256864 22049084

[B28] Situ D Wang J Shao W Assessment and treatment of cancer pain: from Western to Eastern Ann Palliat Med 2011 2011 Oct 6 10.3978/j.issn.22245820.2011.10.01 25841428

[B29] Luo RJ Zhang DD Zhu J Association study on single nucleotide polymorphisms in HTRA1 gene and rheumatoid arthritis Zhonghua Yi Xue Yi Chuan Xue Za Zhi 2011 27 305 309 20533271 10.3760/cma.j.issn.1003-9406.2010.0.015

[B30] Shiga A Nozaki H Yokoseki A Cerebral small-vessel disease protein HTRA1 controls the amount of TGF-beta1 via cleavage of proTGF-beta1 Hum Mol Genet 2011 20 1800 1810 10.1093/hmg/ddr063 21320870

[B31] Marzioni D Quaranta A Lorenzi T Expression pattern alterations of the serine protease HtrA1 in normal human placental tissues and in gestational trophoblastic diseases Histol Histopathol 2009 24 1213 1222 19688690 10.14670/HH-24.1213

[B32] Bowden MA Di Nezza-Cossens LA Jobling T Serine proteases HTRA1 and HTRA3 are down-regulated with increasing grades of human endometrial cancer Gynecol Oncol 2006 103 253 260 10.1016/j.ygyno.2006.03.006 16650464

[B33] Narkiewicz J Klasa-Mazurkiewicz D Zurawa-Janicka D Changes in mRNA and protein levels of human HtrA1, HtrA2 and HtrA3 in ovarian cancer Clin Biochem 2008 41 561 569 10.1016/j.clinbiochem.2008.01.004 18241672

[B34] De Luca A De Falco M Severino A Distribution of the serine protease HtrA1 in normal human tissues J Histochem Cytochem 2003 51 1279 1284 10.1177/002215540305101004 14500695

[B35] Mullany SA Moslemi-Kebria M Rattan R Expression and functional significance of HtrA1 loss in endometrial cancer Clin Cancer Res 2010 17 427 436 21098697 10.1158/1078-0432.CCR-09-3069 PMC3057564

[B36] Chien J Ota T Aletti G Serine protease HtrA1 associates with microtubules and inhibits cell migration Mol Cell Biol 2009 29 4177 4187 10.1128/MCB.00035-09 19470753 PMC2715801

[B37] Narkiewicz J Lapinska-Szumczyk S Zurawa-Janicka D Expression of human HtrA1, HtrA2, HtrA3 and TGF-beta1 genes in primary endometrial cancer Oncol Rep 2009 21 1529 1537 19424634 10.3892/or_00000385

[B38] Launay S Maubert E Lebeurrier N HtrA1-dependent proteolysis of TGF-beta controls both neuronal maturation and developmental survival Cell Death Differ 2008 15 1408 1416 10.1038/cdd.2008.82 18551132

